# Correction: Pneumococcal vaccination at 65 years and vaccination coverage in at-risk adults: A retrospective population-based study in France

**DOI:** 10.1371/journal.pone.0350226

**Published:** 2026-05-26

**Authors:** Benjamin Wyplosz, Benjamin Grenier, Nicolas Roche, François Roubille, Paul Loubet, Ariane Sultan, Bertrand Fougère, Jérôme Fernandes, Didier Duhot, Bruno Moulin, Fanny Raguideau, Emmanuelle Blanc, Gwenael Goussiaume

In Fig 2, an error in the vaccine coverage rate numbers was identified. The influenza vaccination rate for all patients was incorrectly stated as 46.2% in Fig 2A. The correct rate is 53.8%. Additionally, for the diabetic patients, the influenza vaccination rate was incorrectly stated as 44.5%; the correct rate is 55.6%. Please see the correct Fig 2 here. The corrections do not change the underlying conclusions of the study.

The vaccine coverage rates for France are calculated differently than the rates in the manuscript. Please view the correct S1 File below.

**Fig 2:**
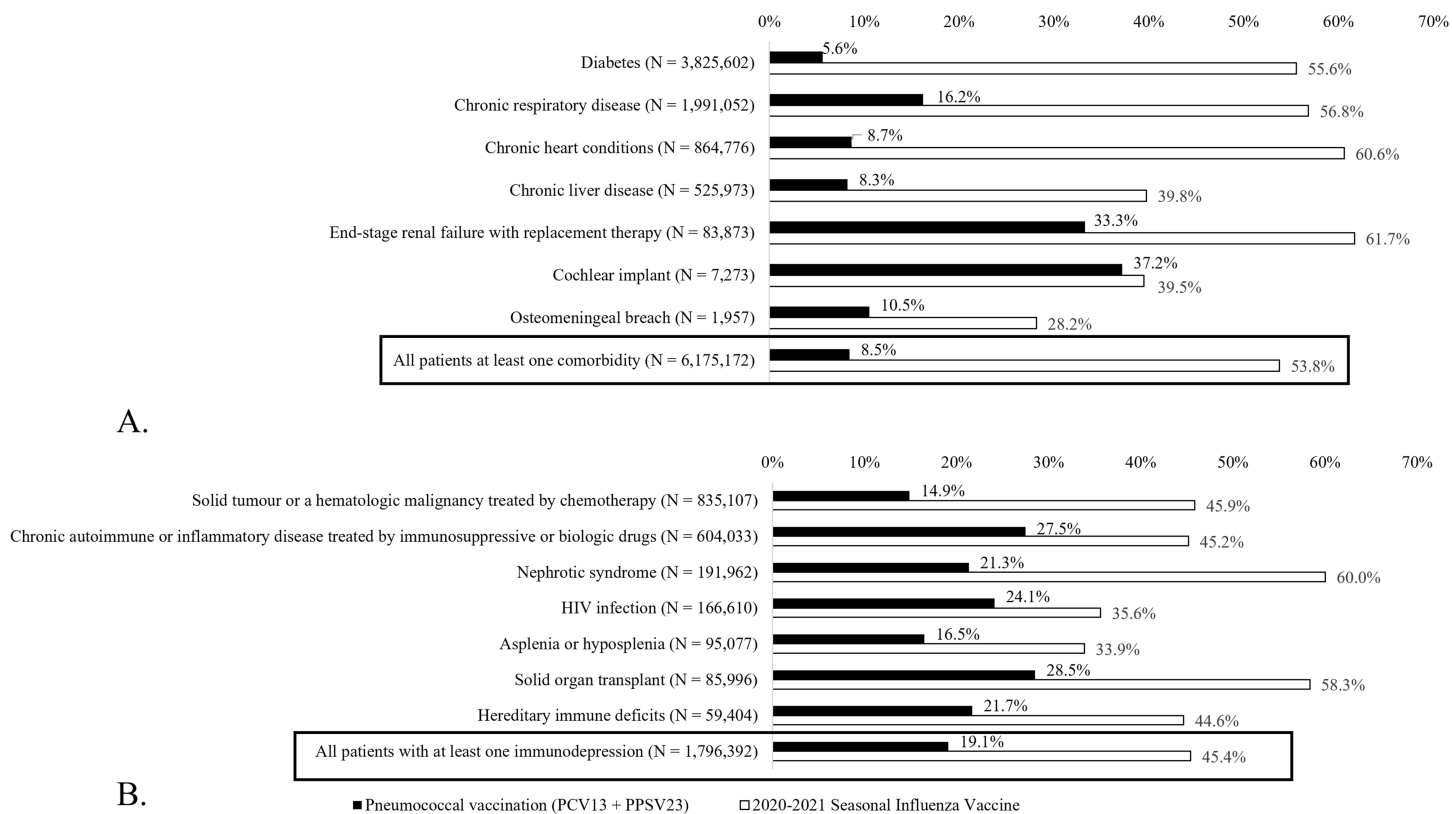
Pneumococcal (black bars) and influenza (white bars) vaccine coverage in patients living with comorbidities (A), and immunosuppressive conditions (B), who are at risk of pneumococcal disease in France in 2020. Populations are listed in descending order of numerical value.

## Supporting information

S1 File Vaccine coverage rate per region. (XLSX)
