# Supplementary material for: Pneumococcal vaccination at 65 years and vaccination coverage in at-risk adults: A retrospective population-based study in France
Source: PLoS One. 2025 Aug 11;20(8):e0329703. doi: 10.1371/journal.pone.0329703 (PMC12338810; doi:10.1371/journal.pone.0329703)
Supplement: S1 Methods — (DOCX) [file pone.0329703.s001.docx]

## **S1 Methods. Algorithms to identify the cohorts**

| Population | Algorithm | ICD-10 codes | CCAM code | ATC Class | GHM | LPP codes |
| --- | --- | --- | --- | --- | --- | --- |
| Asplenic or hyposplenic patients | Long-Term Disease (LTD) from 2009 to year N (i.e. December 12 from study year) OR Principal Diagnosis (PD), Related Diagnosis (RD), or Significant Associated Diagnosis (SAD) within a hospital stay in a Medicine Surgery Obstetrics (MCO) hospital from 2009 to year N OR Medical classification for Clinical Procedure (CCAM) code within a hospital stay in a MCO hospital from 2009 to year N | D730, D73, Q890, D561 , D570 , D572 , C261 , P151 , S360 , Q890 , Z8502 | FFFC420, FFFA002, FFFC001, FFFA001, FFQX005, HNQX007, HEPA004, HEPA007, HNFC002, HNFA013, HNFA010, HNFA006, HNFA004 |  |  |  |
| Hereditary immune deficits affected patients | LTD during year N OR PD, RD, or SAD within a hospital stay in a MCO hospital during year N | D81, D82, D83, D84, D86, D89, G113 |  |  |  |  |
| HIV patients | Cnam pathologies mapping (G8 version) [1] : top_IRVih_ind |  |  |  |  |  |
| Chemotherapy-treated solid cancer or hematologic malignancy affected patients | Chemotherapy noted as PD/RD within a Medical Unit of a stay in a MCO hospital during year N OR At least 3 different dispensing dates for oral chemotherapy (community or outpatient pharmacy “*Rétrocession”* setting) during year N | Z511, Z512, Z082 | L01 |  |  |  |
| Solid organ transplanted patients | LTD during year N OR PD, RD, or SAD within a hospital stay in a MCO hospital from 2009 to year N OR Diagnosis-Related Group (DRG) within a hospital stay in a MCO hospital from 2009 to year N OR CCAM code within a hospital stay in a MCO hospital from 2009 to year N OR At least 3 different dispensing dates for anti-rejection drug (community setting) during year N | Z940 , Z941 , Z942 , Z943 , Z944 , Z9481 , Z9482 | JAEA003, JAEA002, DZEA002, DZEA003, DZEA001, DZEA004, GFEA005, GFEA002, GFEA003, GFEA007, GFEA004, GFEA001, GFEA006, HGEA005, HGEA002, HGEA004, HLEA002, HLEA001, HNEA900, HNEA002, HNEH900 | L04AA06, L04AA10, L04AA18 | 27C061, 27C062, 27C063, 27C064, 11M171, 11M172, 11M173, 11M174 |  |
| Patients affected by chronic autoimmune or inflammatory diseases treated by immunosuppressive or biologic drugs | Cnam pathologies mapping (G8 version) [1]: top_IRCrRCH_ind (Crohn and Ulcerative Colitis) OR top_IRPolyA_ind (Rheumatoid Arthritis, RA) OR top_IRSponA_ind (Ankylosing spondyloarthritis, AS) OR top_IRautre_ind AND  At least 3 different dispensing dates for immunosuppressive or biologic drugs (community and expensive drugs lists inpatient/outpatient setting) during years N-1 to N |  |  | H02AB01, H02AB02, H02AB04, H02AB05, H02AB06, H02AB07, H02AB08, H02AB09, H02AB10, H02AB13, H02AB17, L04AA02, L04AA03, L04AA04, L04AA06, L04AA10, L04AA13, L04AA18, L04AA21, L04AA23, L04AA24, L04AA25, L04AA26, L04AA27, L04AA28, L04AA29, L04AA31, L04AA32, L04AA33, L04AA34, L04AA36, L04AA37, L04AA44, L04AB01, L04AB02, L04AB04, L04AB05, L04AB06, L04AC01, L04AC02, L04AC03, L04AC04, L04AC05, L04AC07, L04AC08, L04AC10, L04AC11, L04AC12, L04AC13, L04AC14, L04AC16, L04AC17, L04AC18, L04AD01, L04AD02, L04AX01, L04AX02, L04AX03, L04AX04, L04AX05, L04AX06, L04AX07, L04AX08 |  |  |
| Nephrotic syndrome affected patients | Nephrotic syndrome affected patients LTD during year N OR Nephrotic syndrome PD, RD, or SAD within a hospital stay in a MCO hospital from years N-5 to year N | N04, N184, N185 |  |  |  |  |
| Chronic respiratory disease (COPD, emphysema, bronchus and lung cancer, Interstitial pneumonia, Cystic fibrosis and non-cystic fibrosis bronchiectasis, pulmonary hypertension, or Severe asthma under continuous treatment) | LTD during year N OR PD/RD within a Medical Unit of a stay in a MCO hospital during years N-5 to N OR SAD within a hospital stay in a MCO hospital during year N OR ( Age > 40 years during year N AND Absence of inhaled corticosteroids dispensing during year N AND Absence of montelukast dispensing during year N AND At least 2 dispensing dates for Long-Acting Beta Agonists during year N )  At least 1 Omalizumab dispensing during year N  OR  (  (Asthma LTD without COPD LTD during year N  OR  Asthma PD or RD without COPD PD, RD, or SAD within a hospital stay in a MCO hospital from year N-5 to year N)  ET  At least 10 dispensing date for severe asthma treatments during year N (ATC class: R03AC, R03AK, R03BA)  ) | J40, J41, J42, J43, J44, J45, J46, J960, J961, J60, J61, J63, J64, J65, J66, J67, J684, J701, J702, J703, J82, J84 |  | R03AC12, R03AC13, R03AC18, R03AC19, R03AK06, R03AK08, R03AL03, R03AL04, R03AL06, R03AL08, R03AL09, R03BB04, R03BB06, R03BB07, R03BA01, R03BA02, R03BA03, R03BA05, R03DC03, R03DX05, R03AC12, R03AC13, R03AC18, R03AC19, R03AK06, R03AK07, R03AK08, R03AK10, R03AK11, R03BA01, R03BA02, R03BA03, R03BA05, R03BA07, R03BA08 |  |  |
| Cyanotic heart disease | PD, RD, or SAD within a hospital stay in a MCO hospital from years 2009 to N | Q20, Q21, Q22, Q23, Q24, Q25 |  |  |  |  |
| Heart failure | Cnam pathologies mapping (G8 version) [1]: top_CvIC_aig + top_CvIC_chr |  |  |  |  |  |
| Patients end-stage renal failure with replacement therapy | Cnam pathologies mapping (G8 version) [1]: sup_RIRCT_cat (top_RDialyse_ind + top_Rtrans_aig + top_Rtrans_chr) without patients identification of dialysis in FCR hospitals only |  |  |  |  |  |
| Chronic liver disease | Cnam pathologies mapping (G8 version) [1]: adapted from top_HFoiPan_ind (pancreatic diseases were removed, ICD-10 codes K85 and K86) |  |  |  |  |  |
| Treated diabetes | Cnam pathologies mapping (G8 version) [1]: top_FDiabet_ind |  |  |  |  |  |
| Osteomeningeal breach | PD, RD, or SAD within a hospital stay in a MCO hospital during year N OR CCAM code within a hospital stay in a MCO hospital during year N  Patients are considered at-risk for a maximum duration of 1 year after breach identification. Follow-up is stopped after this period. | G960 | ABFA007 , ABSA001 , ABSA002 , ABSA003 , ABSA004 , ABSA005 , ABSA006 , ABSA007 , ABSA008 , ABSA009 , ABSA010 , ABSA011 , ABSA012 |  |  |  |
| Cochlear implant | List of products and services qualifying for reimbursement (LPPR) code within a MCO hospital from 2009 to year N |  |  |  |  | 3421417, 3463686, 3408121, 3443212, 3458797, 3477872, 3471585, 3449835, 3494215, 3415368, 3451192, 3453297, 3419892, 3400869, 3403299, 3485950, 3415960, 3453357, 3401188, 3434609, 3446720, 3471148, 3415345, 3444163, 3444269, 3473791, 3434302, 3419515, 3436123, 3401277, 3416095, 3471349 |
