# Supplementary material for: Pneumococcal vaccination at 65 years and vaccination coverage in at-risk adults: A retrospective population-based study in France
Source: PLoS One. 2025 Aug 11;20(8):e0329703. doi: 10.1371/journal.pone.0329703 (PMC12338810; doi:10.1371/journal.pone.0329703)
Supplement: S1 Table — (DOCX) [file pone.0329703.s002.docx]

## **S1 Table. Characteristics of the patients with a comorbidity associated with an increased risk of pneumococcal disease in France in 2020.**

|  | Diabetes  N = 3,825,602 | Chronic respiratory disease  N = 1,991,052 | Heart failure  N = 763,945 | Chronic liver disease  N = 525,973 | Cyanotic heart disease  N = 100,831 | End-stage renal failure with replacement therapy  N = 83,873 | Cochlear implant  N = 7,273 | Osteomeningeal breach  N = 1,957 | Total  N = 6,175,172 |
| --- | --- | --- | --- | --- | --- | --- | --- | --- | --- |
| Age |  |  |  |  |  |  |  |  |  |
| Mean (SD) | 67.1 (13.4) | 67.5 (14.2) | 76.7 (13.2) | 59.0 (15.4) | 54.6 (18.4) | 63.6 (15.1) | 59.3 (18.4) | 54.8 (16.5) | 66.6 (14.6) |
| 18-45 years, n (%) | 249,109 (6.5) | 131,788 (6.6) | 17,925 (2.4) | 102,032 (19.4) | 31,903 (31.6) | 11,226 (13.4) | 1,662 (22.9) | 576 (29.4) | 509,521 (8.3) |
| 46-65 years, n (%) | 1,335,321 (34.9) | 703,605 (35.3) | 125,714 (16.5) | 236,793,(45.0) | 36,890 (36.6) | 30,767 (36.7) | 2,377 (32.7) | 792 (40.5) | 2,167,107 (35.1) |
| 66-75 years, n (%) | 1,226,105 (32.1) | 559,571 (28.1) | 173,975 (22.8) | 114,185,(21.7) | 18,864 (18.7) | 22,644 (27.0) | 1,757 (24.2) | 398 (20.3) | 1,776,264 (28.8) |
| 76-85 years, n (%) | 744,151 (19.5) | 394,243 (19.8) | 222,513 (29.1) | 54,327 (10.3) | 10,064 (10.0) | 14,514 (17.3) | 1,177 (16.2) | 170 (8.7) | 1,159,921 (18.8) |
| >85 years, n (%) | 270,916 (7.1) | 201,845 (10.1) | 223,818 (29.3) | 18,636 (3.5) | 3,110 (3.1) | 4,722 (5.6) | 300 (4.1) | 21 (1.1) | 562,359 (9.1) |
| Women, n (%) | 1,703,019 (44.5) | 919,549 (46.2) | 350,222 (45.8) | 226,147 (43.0) | 43,954 (43.6) | 32,098 (38.3) | 4,139 (56.9) | 1,003 (51.3) | 2,827,988 (45.8) |
| Hospitalisations |  |  |  |  |  |  |  |  |  |
| Patients with at least one encounter in 2020, n (%) | 1,089,838 (28.5) | 842,250 (42.3) | 416,932 (54.6) | 261,767 (49.8) | 36,654 (36.4) | 65,806 (78.5) | 2,416 (33.2) | 1,957 (100.0) | 2,071,676 (33.6) |
| Median (IQR) number of encounter in 2020 | 0.0 (1.0) | 0.0 (1.0) | 1.0 (2.0) | 0.0 (2.0) | 0.0 (1.0) | 5.0 (26.0) | 0.0 (1.0) | 2.0 (2.0) | 0.0 (1.0) |
| General practitioner |  |  | S |  |  |  |  |  |  |
| Patients with at least one encounter in 2020, n (%) | 3,374,467 (88.2) | 1,718,471 (86.3) | 626,248 (82.0) | 442,149 (84.0) | 85,449 (84.7) | 59,375 (70.8) | 6,186 (85.1) | 1,735 (88.7) | 5,345,506 (86.6) |
| Median (IQR) number of encounter in 2020 | 5.0 (5.0) | 5.0 (6.0) | 6.0 (7.0) | 5.0 (7.0) | 4.0 (5.0) | 2.0 (6.0) | 4.0 (5.0) | 6.0 (6.0) | 5.0 (5.0) |
| Private specialist physician |  |  |  |  |  |  |  |  |  |
| Patients with at least one encounter in 2020, n (%) | 1,849,155 (48.3) | 985,416 (49.5) | 360,086 (47.1) | 245,338 (46.6) | 50,341 (49.9) | 50,586 (60.3) | 3,567 (49.4) | 1,291 (66.0) | 2,976,109 (48.2) |
| Median (IQR) number of encounter in 2020 | 0.0 (2.0) | 0.0 (2.0) | 0.0 (2.0) | 0.0 (2.0) | 0.0 (2.0) | 1.0 (4.0) | 0.0 (2.0) | 1.0 (4.0) | 0.0 (2.0) |
| Community nurse |  |  |  |  |  |  |  |  |  |
| Patients with at least one encounter in 2020, n (%) | 3,041,229 (79.5) | 1,498,591 (75.3) | 639,948 (83.8) | 384,798 (73.2) | 68,487 (67.9) | 65,423 (78.0) | 4,716 (64.8) | 1,695 (86.6) | 4,738,226 (76.7) |
| Median (IQR) number of encounter in 2020 | 3.0 (6.0) | 2.0 (11.0) | 6.0 (7.0) | 7.0 (58.0) | 1.0 (5.0) | 6.0 (36.0) | 1.0 (4.0) | 7.0 (17.0) | 2.0 (7.0) |
