# Supplementary material for: Pneumococcal vaccination at 65 years and vaccination coverage in at-risk adults: A retrospective population-based study in France
Source: PLoS One. 2025 Aug 11;20(8):e0329703. doi: 10.1371/journal.pone.0329703 (PMC12338810; doi:10.1371/journal.pone.0329703)
Supplement: S2 Table — (DOCX) [file pone.0329703.s003.docx]

## **S2 Table. Characteristics of the immunocompromised patients in France in 2020.**

|  | Chemotherapy-treated solid cancer or hematologic malignancy  N = 835,107 | Chronic autoimmune or inflammatory disease treated by immunosuppressive or biologic drugs  N = 604,033 | Nephrotic syndrome  N = 191,962 | HIV  N = 166,190 | Asplenia or hyposplenia  N = 95,077 | | Solid organ transplant  N = 85,996 | | Hereditary immune deficits  N = 59,404 | Total  1,796,392 | |
| --- | --- | --- | --- | --- | --- | --- | --- | --- | --- | --- | --- |
| Age |  |  |  |  |  | |  | |  |  | |
| Mean (SD) | 60.2 (17.1) | 58.8 (17.4) | 70.2 (16.9) | 50.3 (12.9) | 52.5 (19.4) | 57.1 (14.8) | | 57.1 (16.3) | | 59.8 (17.5) |  |
| 18-45 years, n (%) | 179,459 (21.5) | 144,544 (23.9) | 18,966 (9.9) | 57,707 (34.7) | 37,826 (39.8) | 18,963 (22.1) | | 15,016 (25.3) | | 402,542 (22.4) |  |
| 46-65 years, n (%) | 284,337 (34.0) | 223,786 (37.1) | 44,437 (23.2) | 89,658 (54.0) | 28,561 (30.0) | 39,281 (45.7) | | 24,376 (41.0) | | 643,706 (35.8) |  |
| 66-75 years, n (%) | 209,609 (25.1) | 123,880 (20.5) | 45,582 (23.8) | 14,654 (8.8) | 15,890 (16.7) | 20,325 (23.6) | | 12,104 (20.4) | | 393,621 (21.9) |  |
| 76-85 years, n (%) | 125,497 (15.0) | 82,097 (13.6) | 46,121 (24.0) | 3,663 (2.2) | 9,463 (10.0) | 6,618 (7.7) | | 6,162 (10.4) | | 254,048 (14.1) |  |
| >85 years, n (%) | 36,856 (4.3) | 29,726 (4.9) | 36,205 (4.3) | 508 (0.3) | 3,337 (3.5) | 809 (0.9) | | 1,746 (2.9) | | 102,475 (5.7) |  |
| Women, n (%) | 499,832 (59.9) | 381,475 (63.2) | 82,087 (42.8) | 56,054 (33,7) | 46,549 (49.0) | 35,567 (41.4) | | 31,754 (53.5) | | 1,005,28 (56.0) |  |
| Hospitalisations |  |  |  |  |  |  | |  | |  |  |
| Patients with at least one encounter in 2020, n (%) | 592,266 (70.9) | 248,136 (41.1) | 130,901 (68.2) | 43,811 (26.4) | 39,661 (41.7) | 51,491 (59.9) | | 34,464 (58.0) | | 955,484 (53.2) |  |
| Median (IQR) number of encounter in 2020 | 2.0 (9.0) | 0.0 (1.0) | 2.0 (6.0) | 0.0 (1.0) | 0.0 (1.0) | 1.0 (3.0) | | 1.0 (3.0) | | 1.0 (3.0) |  |
| General practitioner |  |  |  |  |  |  | |  | |  |  |
| Patients with at least one encounter in 2020, n (%) | 722,677 (86.5) | 525,792 (87.0) | 147,254 (76.7) | 125,151 (75.3) | 76,059 (80.0) | 66,006 (76.8) | | 50,600 (85.2) | | 1,514,578 (84.3) |  |
| Median (IQR) number of encounter in 2020 | 5.0 (7.0) | 5.0 (7.0) | 4.0 (8.0) | 3.0 (5.0) | 4.0 (6.0) | 5.0 (5.0) | | 5.0 (7.0) | | 5.0 (6.0) |  |
| Private specialist physician |  |  |  |  |  |  | |  | |  |  |
| Patients with at least one encounter in 2020, n (%) | 499,607 (59.8) | 395,046 (65.4) | 101,794 (53.0) | 57,472 (34.6) | 40,073 (42.2) | 45,607 (53.0) | | 30,576 (51.5) | | 1,034,606 (57.6) |  |
| Median (IQR) number of encounter in 2020 | 1.0 (3.0) | 1.0 (4.0) | 1.0 (3.0) | 0.0 (1.0) | 0.0 (2.0) | 1.0 (2.0) | | 1.0 (2.0) | | 1.0 (3.0) |  |
| Community nurse |  |  |  |  |  |  | |  | |  |  |
| Patients with at least one encounter in 2020, n (%) | 728,053 (87.2) | 491,310 (81.3) | 157,316 (82.0) | 96,414 (58.0) | 62,372 (65.6) | 70,280 (81.7) | | 46,628 (78.5) | | 1,447,679 (80.6) |  |
| Median (IQR) number of encounter in 2020 | 7.0 (26.0) | 3.0 (8.0) | 10.0 (75.0) | 1.0 (2.0) | 1.0 (8.0) | 4.0 (13.0) | | 3.0 (12.0) | | 4.0 (16.0) |  |
| HIV: human immunodeficiency virus, SD: Standard deviation, FDep: French DEPrivation index, IQR: Interquartile Range. | | | | | | | | | | | |
