# Supplementary material for: Pneumococcal vaccination at 65 years and vaccination coverage in at-risk adults: A retrospective population-based study in France
Source: PLoS One. 2025 Aug 11;20(8):e0329703. doi: 10.1371/journal.pone.0329703 (PMC12338810; doi:10.1371/journal.pone.0329703)
Supplement: S3 Table — (DOCX) [file pone.0329703.s004.docx]

## **S3** **Table. Characteristics associated with pneumococcal primary vaccination (PCV-13 + PPV 23) and 2020-2021 Seasonal Influenza vaccination in patients with comorbidities in France in 2020 (multivariable results).**

|  | Model 1: Pneumococcal primary vaccination (PCV-13 + PPV 23) | | | Model 2: 2020-2021 Seasonal Iinfluenza vaccination | | |
| --- | --- | --- | --- | --- | --- | --- |
| Variable | N patients / N total patients (%) | Odds ratio (95%CI) | p-value | N patients / N total patients (%) | Odds ratio (95% CI) | p-value |
| Comorbidities |  |  |  |  |  |  |
| Sex |  |  | <.0001 |  |  | <.0001 |
| Male | 285,012/3,347,184 (8.5) | Ref. |  | 1,824,691/3,347,184 (54.5) | Ref. |  |
| Female | 237,056/2,827,988 (8.4) | 0.984 [0.978;0.989] |  | 1,500,243/2,827,988 (53.0) | 0.837 [0.834;0.840] |  |
| Age (years) |  |  | <.0001 |  |  | <.0001 |
| 18-65 | 193,263/2,676,628 (7.2) | Ref. |  | 950,473/2,676,628 (35.5) | Ref. |  |
| > 65 | 328,805/3,498,544 (9.4) | 1.184 [1.177;1.191] |  | 2,374,461/3,498,544 (67.9) | 3.551 [3.539;3.564] |  |
| At risk conditions (N) |  |  | <.0001 |  |  | <.0001 |
| 1 | 293,552/4,763,008 (6.2) | Ref. |  | 2,446,044/4,763,008 (51.4) | Ref. |  |
| 2 | 146,237/1,040,725 (14.1) | 2.258 [2.243;2.274] |  | 637,687/1,040,725 (61.3) | 1.253 [1.247;1.259] |  |
| ≥ 3 | 82,012/368,327 (22.3) | 3.747 [3.711;3.783] |  | 240,452/368,327 (65.3) | 1.487 [1.475;1.499] |  |
| Visits to a general practitioner in 2020 (N) |  |  | <.0001 |  |  | <.0001 |
| 0 | 62,382/829,666 (7.5) | Ref. |  | 368,213/829,666 (44.4) | Ref. |  |
| 1-4 | 233,007/3,165,657 (7.4) | 0.977 [0.968;0.986] |  | 1,640,910/3,165,657 (51.8) | 1.266 [1.260;1.273] |  |
| ≥5 | 226,679/2,179,849 (10.4) | 1.428 [1.415;1.441] |  | 1,315,811/2,179,849 (60.4) | 1.523 [1.514;1.532] |  |
| Visits to a private specialist physician in 2020 (N) |  |  | <.0001 |  |  | <.0001 |
| 0 | 222,157/3,199,063 (6.9) | Ref. |  | 1,543,976/3,199,063 (48.3) | Ref. |  |
| 1-4 | 231,784/2,423,680 (9.6) | 1.417 [1.408;1.426] |  | 1,437,534/2,423,680 (59.3) | 1.368 [1.363;1.373] |  |
| ≥5 | 68,127/552,429 (12.3) | 1.885 [1.868;1.902] |  | 343,424/552,429 (62.2) | 1.465 [1.455;1.474] |  |
| Visits to a community nurse in 2020 (N) |  |  | <.0001 |  |  | <.0001 |
| 0 | 84,408/1,436,946 (5.9) | Ref. |  | 502,208/1,436,946 (34.9) | Ref. |  |
| 1-4 | 207,751/2,660,844 (7.8) | 1.357 [1.346;1.368] |  | 1,456,071/2,660,844 (54.7) | 1.987 [1.978;1.995] |  |
| 5+ | 229,909/2,077,382 (11.1) | 1.994 [1.978;2.010] |  | 1,366,655/2,077,382 (65.8) | 2.466 [2.454;2.479] |  |
| Hospital admissions in 2020 (N) |  |  | <.0001 |  |  | <.0001 |
| 0 | 289,911/4,103,496 (7.1) | Ref. |  | 2,117,451/4,103,496 (51.6) | Ref. |  |
| 1-4 | 184,028/1,810,260 (10.2) | 1.489 [1.479;1.498] |  | 1,051,564/1,810,260 (58.1) | 0.885 [0.881;0.889] |  |
| ≥5 | 48,129/261,416 (18.4) | 2.970 [2.938;3.001] |  | 155,919/261,416 (59.6) | 0.728 [0.721;0.735] |  |
| Immunocompromised patients |  |  |  |  |  |  |
| Sex |  |  | <.0001 |  |  | <.0001 |
| Male | 164,749/791,104 (20.8) | Ref. |  | 392,902/791,104 (49.7) | Ref. |  |
| Female | 178,522/1,005,288 (17.8) | 0.841 [0.835;0.847] |  | 426,751/1,005,288 (42.5) | 0.745 [0.740;0.750] |  |
| Age (years) |  |  | <.0001 |  |  | <.0001 |
| 18-65 | 208,874/1,046,248 (20.0) | Ref. |  | 285,621/1,046,248 (27.3) | Ref. |  |
| > 65 | 134,397/750,144 (17.9) | 0.753 [0.746;0.759] |  | 534,032/750,144 (71.2) | 5.473 [5.435;5.511] |  |
| At risk conditions (N) |  |  | <.0001 |  |  | <.0001 |
| 1 | 170,528/1,053,040 (16.2) | Ref. |  | 391,050/1,053,040 (37.1) | Ref. |  |
| 2 | 103,675/479,885 (21.6) | 1.504 [1.491;1.518] |  | 259,414/479,885 (54.1) | 1.584 [1.572;1.597] |  |
| ≥3 | 69,068/263,467 (26.2) | 2.025 [2.003;2.047] |  | 169,189/263,467 (64.2) | 2.081 [2.059;2.102] |  |
| Visits to a general practitioner in 2020 (N) |  |  | <.0001 |  |  | <.0001 |
| 0 | 49,373/281,814 (17.5) | Ref. |  | 106,250/281,814 (37.7) | Ref. |  |
| 1-4 | 168,694/886,073 (19.0) | 1.043 [1.031;1.055] |  | 376,411/886,073 (42.5) | 1.218 [1.206;1.231] |  |
| ≥5 | 125,204/628,505 (19.9) | 1.045 [1.033;1.059] |  | 336,992/628,505 (53.6) | 1.516 [1.500;1.533] |  |
| Visits to a private specialist physician in 2020 (N) |  |  | <.0001 |  |  | <.0001 |
| 0 | 133,260/761,786 (17.5) | Ref. |  | 309,170/761,786 (40.6) | Ref. |  |
| 1-4 | 151,279/742,518 (20.4) | 1.223 [1.213;1.234] |  | 358,864/742,518 (48.3) | 1.249 [1.240;1.259] |  |
| ≥5 | 58,732/292,088 (20.1) | 1.240 [1.226;1.254] |  | 151,619/292,088 (51.9) | 1.289 [1.276;1.302] |  |
| Visits to a community nurse in 2020 (N) |  |  | <.0001 |  |  | <.0001 |
| 0 | 56,188/348,713 (16.1) | Ref. |  | 90,229/348,713 (25.9) | Ref. |  |
| 1-4 | 119,691/604,340 (19.8) | 1.280 [1.266;1.294] |  | 244,200/604,340 (40.4) | 1.754 [1.736;1.772] |  |
| ≥5 | 167,392/843,339 (19.8) | 1.319 [1.304;1.335] |  | 485,224/843,339 (57.5) | 2.311 [2.287;2.335] |  |
| Hospital admissions in 2020 (N) |  |  | <.0001 |  |  | <.0001 |
| 0 | 161,412/840,908 (19.2) | Ref. |  | 343,419/840,908 (40.8) | Ref. |  |
| 1-4 | 111,459/573,016 (19.5) | 0.864 [0.856;0.871] |  | 277,194/573,016 (48.4) | 0.886 [0.879;0.893] |  |
| ≥5 | 70,400/382,468 (18.4) | 0.739 [0.731;0.747] |  | 199,040/382,468 (52.0) | 0.812 [0.804;0.820] |  |
