# Supplementary material for: Pneumococcal vaccination at 65 years and vaccination coverage in at-risk adults: A retrospective population-based study in France
Source: PLoS One. 2025 Aug 11;20(8):e0329703. doi: 10.1371/journal.pone.0329703 (PMC12338810; doi:10.1371/journal.pone.0329703)
Supplement: S1b Fig — (DOCX) [file pone.0329703.s006.docx]

## **S1b Fig. Pneumococcal vaccine coverage rate in France in 2020 in immunocompromised patients (primary vaccination schedule - PCV13 + PPSV23).**
