# Supplementary material for: Pneumococcal vaccination at 65 years and vaccination coverage in at-risk adults: A retrospective population-based study in France
Source: PLoS One. 2025 Aug 11;20(8):e0329703. doi: 10.1371/journal.pone.0329703 (PMC12338810; doi:10.1371/journal.pone.0329703)
Supplement: S2b Fig — (DOCX) [file pone.0329703.s008.docx]

## **S2b Fig. Influenza vaccine coverage rate in France in 2020-2021 for immunocompromised patients.**
