# Supplementary material for: Pneumococcal vaccination at 65 years and vaccination coverage in at-risk adults: A retrospective population-based study in France
Source: PLoS One. 2025 Aug 11;20(8):e0329703. doi: 10.1371/journal.pone.0329703 (PMC12338810; doi:10.1371/journal.pone.0329703)
Supplement: S1 File — (DOCX) [file pone.0329703.s009.docx]

## **S1 File. Vaccine coverage rate per region**
